# Liver Metastasis: A Rare and Sinister Cause of Shoulder Pain

**DOI:** 10.7759/cureus.71966

**Published:** 2024-10-20

**Authors:** Yogesh Lalmalani, Wai L Moy

**Affiliations:** 1 Internal Medicine, Changi General Hospital, Singapore, SGP; 2 Internal Medicine and General Medicine, Sengkang General Hospital, Singapore, SGP

**Keywords:** atypical presentation shoulder pain, colon cancer liver metastasis, referred pain, shoulder joint pain, solitary liver metastasis

## Abstract

Shoulder pain is a common complaint in the elderly, the majority of which is related to musculoskeletal causes, but it can sometimes be a sign of a more sinister problem such as liver metastasis. This case report is about an elderly lady who presented with right shoulder pain, which turned out to be referred pain from liver metastasis. Clues prompting further evaluation included pain triggered by deep breathing while maintaining full shoulder mobility. This case report serves as a reminder to consider and recognise referred pain as a cause of shoulder pain, especially if range of motion and mobility are not affected.

## Introduction

Shoulder pain comprises the third most common musculoskeletal complaint in the primary care setting, with an estimated prevalence rate between 16 and 26% [1]. While intrinsic causes related to the shoulder joint are the most common, it's important to consider extrinsic factors as well to avoid overlooking significant sources of referred pain. A meticulous history and physical examination can help delineate most aetiologies of shoulder pain, whether they are extrinsic or intrinsic [2]. Important extrinsic causes of shoulder pain include gastrointestinal diseases (such as gallstone disease, liver disease, cancer, peritoneal diseases, or splenic conditions), iatrogenic factors (like liver biopsy or laparoscopy), cardiological problems (including myocardial ischemia and pericarditis), neurological factors (such as cervical radiculopathy, brachial plexopathies, and peripheral nerve injuries), tumours that compress the brachial plexus (such as Pancoast tumours), and rheumatological factors contributing to shoulder pain [3]. Extrinsic diseases typically cause pain that is difficult to localise and is usually not affected by passive and active range of motion. Also, specific tests (e.g., drop arm test, empty can/Jobe test, Hawkins test, and painful arc test) for rotator cuff are usually negative [2]. Liver cancer or abscess should be considered when the patient complains of right shoulder pain aggravated by breathing and accompanied by constitutional symptoms such as weight loss and fever. Here, we present a case of an elderly lady who initially presented with right shoulder pain as her sole symptom, and further diagnostic investigations led to a diagnosis unrelated to her shoulder.

## Case presentation

We present the case of an elderly Chinese lady in her seventies who was admitted to the general medicine ward due to an exacerbation of bronchiectasis. Her past medical history was significant for bronchiectasis with prior non-tuberculous mycobacteria infection more than 10 years ago, glaucoma, and a history of internal haemorrhoids. She had a three-day history of worsening shortness of breath, along with a sore throat and cough. She was managed with antibiotics and symptomatic medications. During a review by the ward team, she also reported right shoulder pain, which had been present for three days. The shoulder pain was brought on by inspiration and was not exacerbated by movement. She was unable to characterise the pain but did not experience diurnal variation or early morning stiffness. There was no history of trauma.

**Figure 1:**
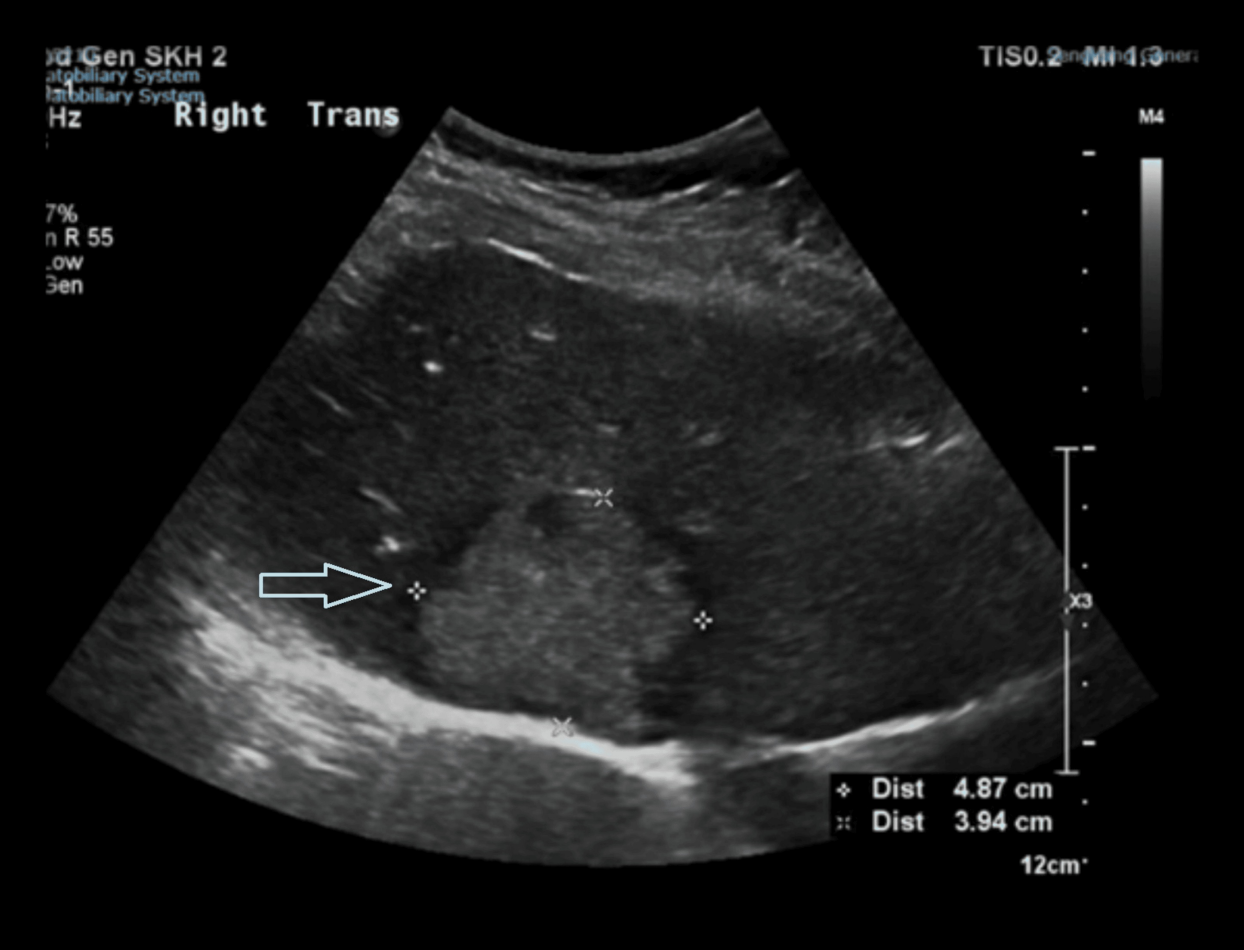
Ultrasound of the liver showing an echogenic lesion in the right hepatic lobe with peripheral hypoechoic halo measuring 4.5 x 4.9 x 3.9 cm

**Figure 2:**
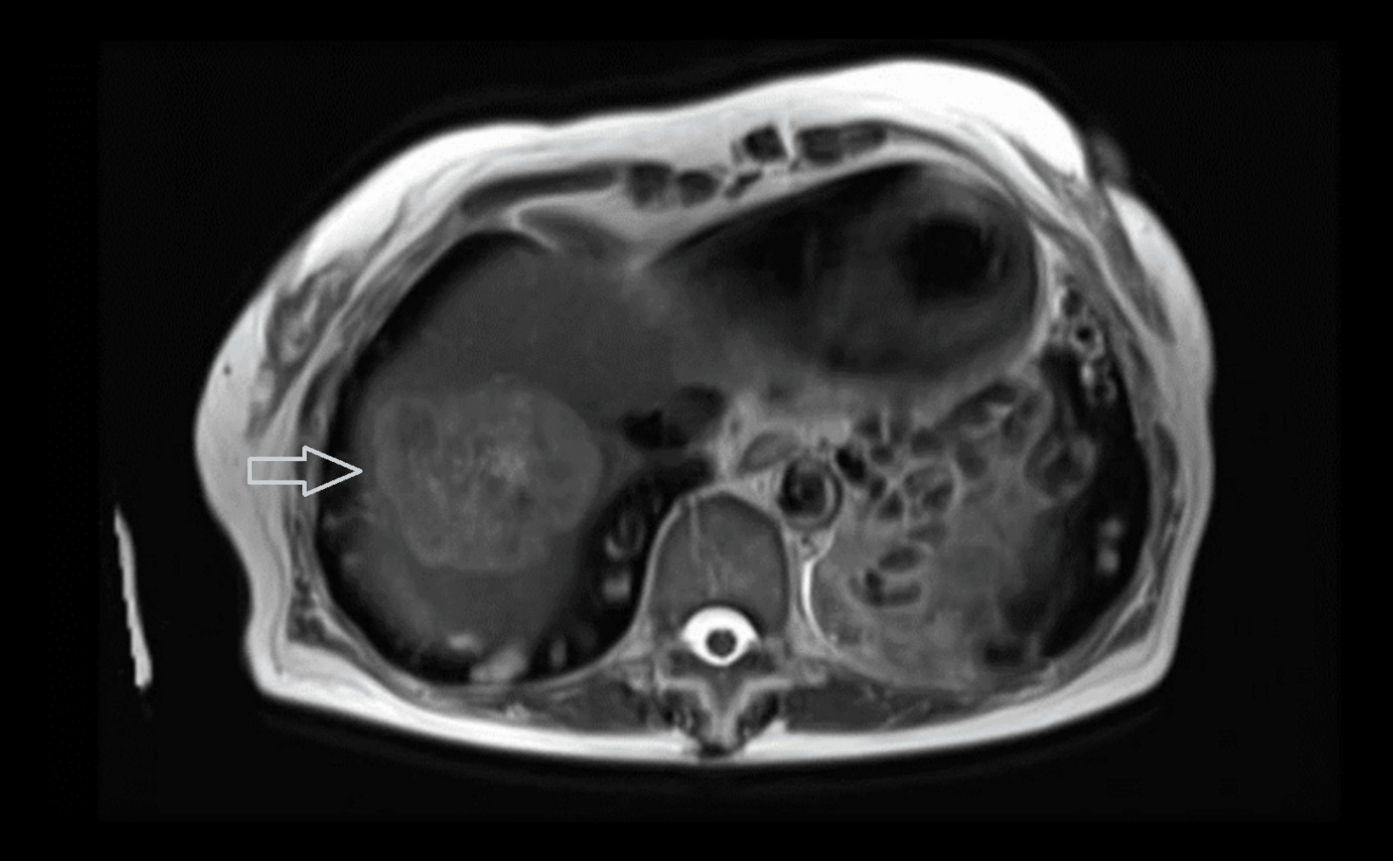
Axial view of MRI liver showing a large 5.2 x4.6 cm mass in the right lobe of liver with patchy high-signal intensity change seen on T2 weighted images

**Figure 3:**
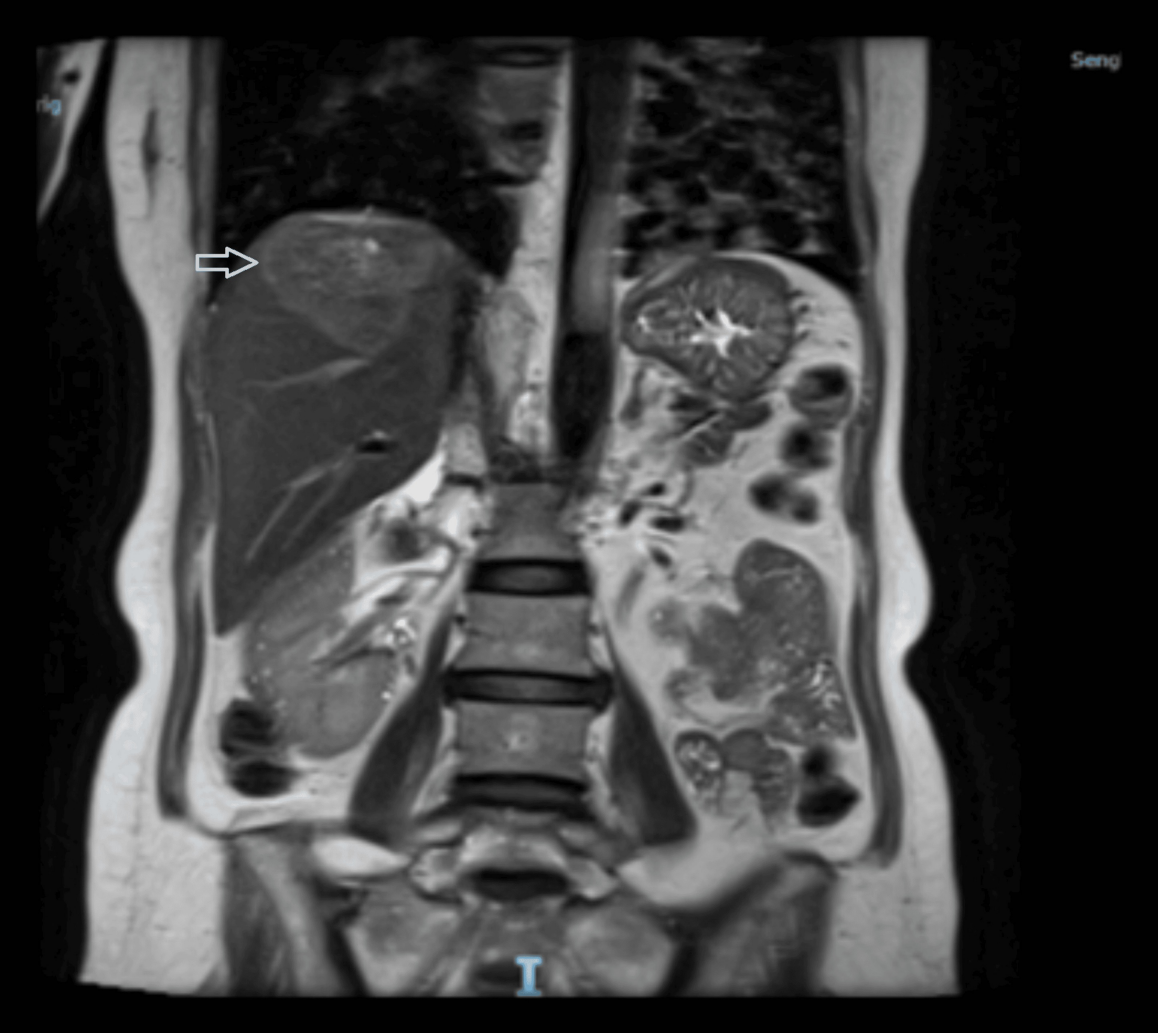
Coronal view of MRI liver showing the liver mass. Note it is located quite close to the diaphragm

On examination, the shoulder joint was unremarkable, with no swelling, warmth, or redness. Both the active and passive ranges of motion of the shoulder were full and painless. The liver function tests upon admission to the hospital were unremarkable (Table 1). We decided to perform an ultrasound of the hepatobiliary system due to the peculiar nature of the pain and suspicion of referred pain. It (Figure 1) revealed a non-specific lobulated echogenic lesion in the right hepatic lobe with a peripheral hypoechoic halo measuring 4.5 x 4.9 x 4.3 cm. A magnetic resonance imaging (MRI) of the liver (Figures 2, 3) was subsequently performed, which showed a large 5.2 x 4.6 cm-sized mass on the right lobe of the liver involving segments 7 and 8, close to the diaphragm with the suggestion of a large central scar tissue, which exhibited patchy high-intensity signal intensity on T2 weighted images. Tumour markers evaluation revealed an elevated serum carcinoembryonic antigen (7.0 μg/L; normal range <= 4.7 μg/L). The CA 19-9 and serum alpha-fetoprotein levels were not raised. The staging computed tomography (CT) of the thorax scan did not reveal any metastasis. She then underwent a CT-guided liver biopsy. The histology revealed moderately differentiated adenocarcinoma, immunoexpression supportive of intestinal-type adenocarcinoma and colon as the most likely primary origin. The patient subsequently underwent a colonoscopy and was found to have a sigmoid colon tumour diagnosed as adenocarcinoma upon histological examination. The patient is now on follow-up with medical oncology for neoadjuvant chemotherapy.

**Table 1:** Liver function test

Parameter	Result (reference range)
Total protein	70 (68-85 g/L)
Albumin	40 (35-50 g/L)
Total bilirubin	6 (< = 21 μmol/L)
Alkaline phosphatase	71 (35-104 U/L)
Alanine transaminase	7 (6-66 U/L)
Aspartate transaminase	19 (12-42 U/L)
Gamma-glutamyl transferase	23 (6-42 U/L)

## Discussion

Referred pain is an important consideration when evaluating shoulder pain. Referred pain can be defined as pain felt at a site remote from the site of origin/stimulation [4]. The mechanism of referred pain is not well understood.

**Figure 4:**
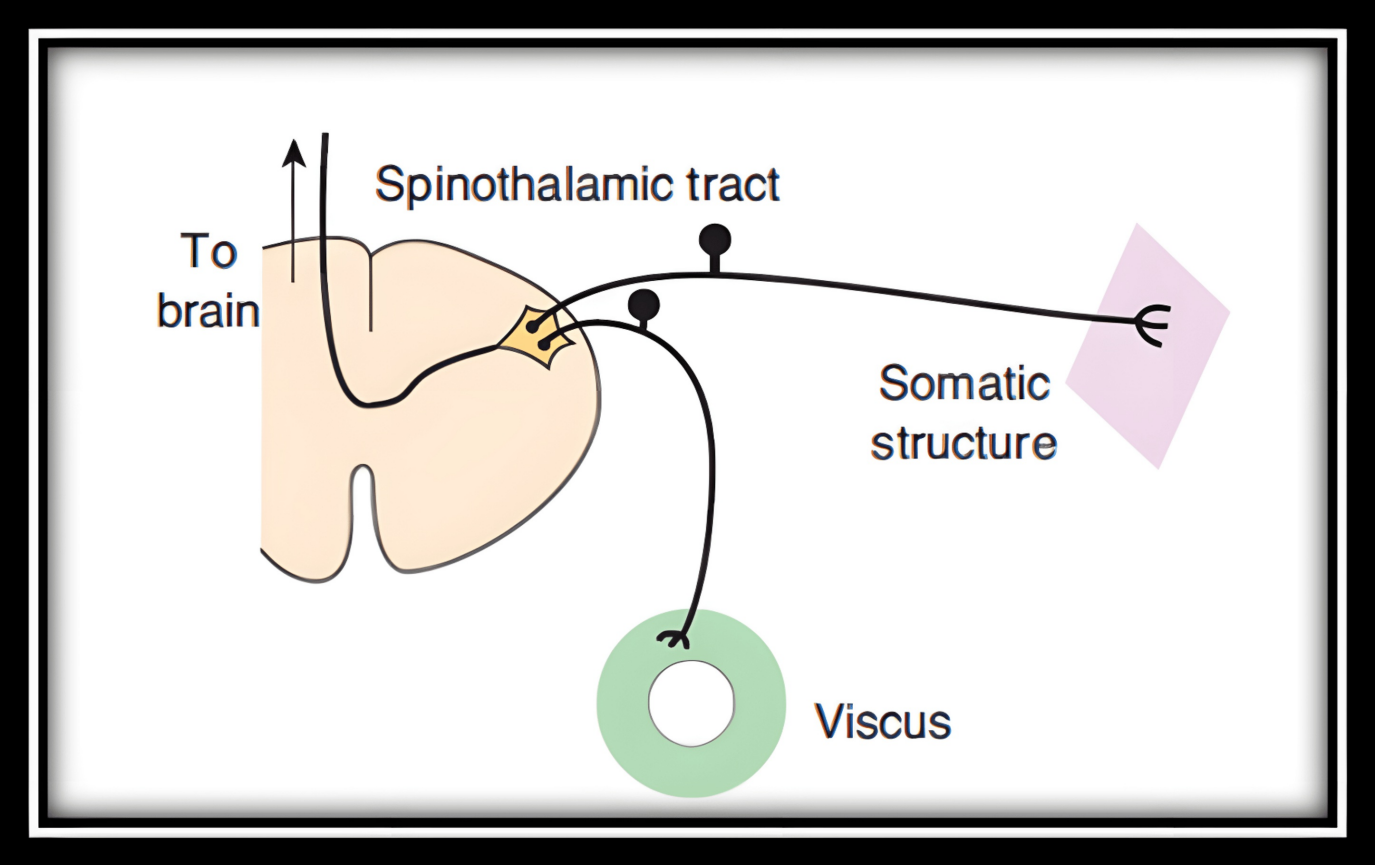
Convergence theory of referred pain Image Credit: McGraw Hill Ganong’s Review of Medical Physiology Edition 23 [7]. This image has been reproduced with permission

One of the more accepted theories of referred pain is the convergence theory, which suggests that the neurons from the somatic structure and the visceral organ both converge onto a single second-order neuron in the ascending tract. When painful stimuli arise in the visceral receptors, the brain is unable to distinguish visceral signals from the signals arising from somatic receptors [5,6]. Figure 4 provides an illustration of the convergence theory of referred pain.

In this patient, we postulate that the irritation of the diaphragm caused by liver metastasis sends out pain signals via the phrenic nerve to the C3-5 spinal levels. The perceived shoulder pain could then be due to either stimulation of the supraclavicular nerves of the cervical plexus (C3-4) that innervate the skin over the shoulder or by stimulation of C-5 articular nerves to the shoulder joint, such as from the axillary or suprascapular nerves. 

In addition to the liver, various structures around the diaphragm can cause referred pain in the shoulder. These include the heart (pericarditis, myocardial ischemia), parietal pleura (lower lobe pneumonia) [8], stomach (gastric perforation) [9], and spleen (splenic perforation with Kehr sign referring to left shoulder pain that is caused by the irritation of the inferior surface of the diaphragm due to bleeding from a splenic rupture). Table 2 summarises common causes of referred pain

**Table 2:** Extrinsic causes of shoulder pain Adapted from Lollino et al., 2012 [3]

Extrinsic causes of shoulder pain	Examples
Gastrointestinal causes	Gallstone disease, Liver disease, Liver cancer or metastasis, Splenic conditions, Peritoneal disease
Cardiological causes	Myocardial ischemia, Pericarditis, Aortic dissection
Neurological causes	Cervical radiculopathy, Brachial plexopathies, Peripheral nerve injuries
Miscellaneous causes	Tumors (e.g.: Pancoast tumor compressing on brachial plexus), Rheumatological causes

Clues indicating the presence of referred pain include signs and symptoms that can be localized to the lungs or gastrointestinal tract. In addition, shoulder pain that is not exacerbated by movement with normal mobility and range of motion is also a characteristic feature. Furthermore, as in our case, pain that is worsened with respiration is also a clue that it could be referred pain. 

## Conclusions

This case report illustrates the importance of including non-musculoskeletal causes of shoulder discomfort in the list of potential differential diagnoses, especially when the physical examination findings do not correlate with the initial presenting symptoms. Physicians should remain vigilant for non-musculoskeletal causes of shoulder pain, particularly when mobility is not impaired. When a patient presents with shoulder pain accompanied by atypical clinical findings, it is wise to resist the temptation of fixating solely on the shoulder as the root cause of discomfort and broaden our perspective beyond shoulder pathology. This case serves as a reminder to explore less common, and potentially sinister, causes of symptoms to ensure a comprehensive and accurate diagnosis.
